# Rapid Electrophoretic Staining and Destaining of Polyacrylamide Gels

**DOI:** 10.3390/mps1020013

**Published:** 2018-04-10

**Authors:** Fumihiro Motojima

**Affiliations:** 1 Biotechnology Research Center and Department of Biotechnology, Toyama Prefectural University, 5180 Kurokawa, Imizu, Toyama 939-0398, Japan; fmotojim@gmail.com; Tel.: +81-766-56-7500; 2 Department of Molecular Bioscience, Kyoto Sangyo University, Kamigamo-Motoyama, Kyoto 603-8555, Japan

**Keywords:** SDS-PAGE, Coomassie brilliant blue, electrophoresis, staining, destaining

## Abstract

Coomassie brilliant blue (CBB) dyes have been commonly used for the staining of protein bands in polyacrylamide gel electrophoresis (PAGE) gels. However, the staining and destaining of CBB dyes are time-consuming, and the use of methanol is hazardous to one’s health. I introduce a rapid electrophoretic destaining method using a semi-dry transfer unit and a high current power supply. In this method, ethanol was used instead of the hazardous methanol. Most of the protein bands became visible in 30 min. After a secondary destaining step, residual CBB was completely destained. The detection limit for a tested protein (5 ng) was higher than that of the conventional method. Therefore, this method is superior in its speed, safety, low cost, and sensitivity.

## 1. Introduction

Polyacrylamide gel electrophoresis (PAGE) is one of the most widely used methods to analyze proteins biochemically [1]. After separation by electrophoresis, protein bands need to be detected with a subsequent staining step. A number of staining methods for PAGE gels have been reported, such as Coomassie brilliant blue (CBB), amido black, and silver staining [2,3,4]. Coomassie brilliant blue staining has been commonly used to stain protein bands in PAGE gels, due to its low cost, easy operation, and relatively high sensitivity [5]. However, according to the typical protocol, the staining and destaining steps require a long time (from 4 h to overnight). Several rapid staining and destaining methods with reduced processing times have been reported. Hervieu reported that CBB-stained sodium dodecyl sulfate (SDS)-PAGE gels could be destained by boiling in distilled water [6]. It has been reported that heat treatment accelerates the staining and destaining steps [7,8]. A modified, rapid Fairbanks Coomassie stain, using a microwave oven, gave a sensitivity limit of 5 ng [9,10]. However, boiling a solution containing methanol is hazardous to one’s health. Dong et al. reported CBB-staining and destaining at boiling temperature without acid and methanol [11]. Lawrence et al. reported a staining method using CBB G-250 with a low concentration of hydrochloric acid, instead of acetic acid [12]. In these procedures, the hot destaining solution should be exchanged repeatedly, leading to risk of burn injury. Kang et al. reported a sensitive colloidal staining method using CBB G-250, using less-toxic ethanol instead of methanol [13,14]. Although this staining method is very sensitive, up to ~1 ng, more than two hours of incubation is required for 80% staining. Several companies produce rapid protein staining/destaining systems, such as eStain™ from GenScript (Piscataway, NJ, USA, Cat. no.: L00657) or Pierce™ Power Stainer from Thermo Scientific (Waltham, MA, USA, Cat. no.: 22840). However, the electrophoretic apparatuses are expensive (~$2000), and each staining costs ~$5 per gel. Here, I introduce a rapid, safe, and less expensive method to stain and destain PAGE gels. In this method, PAGE gels are stained and destained electrophoretically using ethanol instead of methanol. Only one replacement of the boiled water is required. Most of the protein bands became visible in less than 30 min, and thus, it reduces the overall experimental time. Furthermore, the detection limit (5 ng) was lower than that of conventional methods (10 ng).

## 2. Experimental Design

### 2.1 Materials and Methods

#### 2.1.1. Gel Electrophoresis

The ratio of acrylamide and bis-acrylamide was 29:1. The concentration of polyacrylamide in the stacking gel was 5% (*w*/*v*), and the concentration in the separating gel was 15% (*w*/*v*). The running buffer, stacking gel, and separating gel were prepared as previously described [15]. The gels were cast in glass plates for mini gels (Nihon Eido, Tokyo, Japan, Cat. No.: NA1000). The thickness of the gels was 1 mm. The samples were mixed with sample loading buffer prior to loading into the individual wells of the gels. SDS-PAGE was run at 30 mA for ~60 min. 

#### 2.1.2. Conventional CBB Staining and Destaining

The concentrated CBB R-250 solution (5% (*w*/*v*)) was prepared in distilled water and stirred for more than 1 h. The supernatant of the CBB solution, centrifuged at 7000× *g* for 5 min, was used to prepare the staining solution. The gels were agitated in the staining solution containing 0.1% (*w*/*v*) CBB R-250, 50% (*v*/*v*) methanol, and 10% (*v*/*v*) acetic acid at room temperature (~25 °C) for 4 h. Subsequently, the CBB-stained gels were agitated in the destaining solution containing 40% (*v*/*v*) methanol and 10% (*v*/*v*) acetic acid. After several replacements of the destaining solution, the gel was agitated in the destaining solution with 0.0001% (*w*/*v*) CBB R-250. 

#### 2.1.3. Quantification of CBB-Stained Bands

The gels were transilluminated by LED light (ATTO, Tokyo, Japan, Cat. No.: 2196160, FLAT-Viewer) and images were captured with a digital camera (PENTAX, Tokyo, Japan, Cat. No.: Q-S1). The band intensity was quantified with the GelAnalyzer function of ImageJ [16].

### 2.2 Experimental Setup

1. A semi-dry transfer unit: The semi-dry transfer unit used in this study had an anode and cathode made of stainless and platinum-coated titanium, respectively (Bio craft, Tokyo, Japan, Cat. No.: BE-320). The carbon electrode may absorb CBB and affect the general use for western blotting.
2. High current power supply: MP-3AP (Major science, Saratoga, CA, USA) was used in this study.
3. Filter paper was cut to the size of a PAGE gel. In this study, 0.9 mm thick filter paper (ATTO, Tokyo, Japan, Cat. No.: CB-09A) was used.
4. The cathode staining solution: 20% (*v*/*v*) ethanol, 10% (*v*/*v*) acetic acid, 0.1 M glycine, and 0.08% (*w*/*v*) CBB-R 250.
5. The anode solution: 20% (*v*/*v*) ethanol, 10% (*v*/*v*) acetic acid, and 0.1 M glycine.
6. The destaining solution: 20% (*v*/*v*) ethanol and 5% (*v*/*v*) acetic acid.

## 3. Procedure

### 3.1. Fixing Protein Bands by Boiling. Time for Completion: ~5 min

1. Immerse a PAGE gel into 50 mL of deionized water in a plastic container, such as a ZipLoc^®^ container (S. C. Johnson, Racine, WI, USA, Cat. No.: small square, 156 × 156 × 57 mm).
2. The lid of the container should be opened to release the pressure from the boiled water.
3. Heat the solution in a microwave oven at 600 W for 60 s to boil the solution and fix the proteins. Extend the heating time if the solution did not boil.
4. Agitate it for more than 2 min to remove SDS and cool by adding water.

### 3.2. Electrophoretic-Staining/Destaining. Time for Completion: ~20 min

1. Soak the filter paper in the cathode staining solution and spread the solution on the cathode of the semi-dry transfer unit with the filter paper, avoiding air bubbles at the interface.
2. Place the soaked filter paper on the cathode.
3. Immerse the fixed PAGE gel in the anode solution for a few seconds and place it on the filter paper. Avoid air bubbles between the filter paper and the gel.
4. Soak a filter paper in the anode solution and place it on the PAGE gel.
5. Spread the anode solution on the anode to avoid air bubbles.
6. Place the anode on the filter paper.
7. Place a weight of ~1 kg on the semi-dry transfer unit for close contact between the filter paper and electrodes.
8. Run at a constant current of 1200 mA for 15 min by using a high current power supply. A higher current generates heat, resulting in bending of the gel and uneven destaining.
9. If the CBB remained in the gel, change the arrangement of gel and the filter paper and run for an extra ~5 min.
10. Wash the semi-dry transfer unit and cool down the electrode before the next staining.

### 3.3. Secondary Destaining. Time for Completion: 30–60 min

1. Heat the gel in the destaining solution in a microwave oven for 40 s and subsequently agitate at room temperature (~25 °C) for 30–60 min for complete destaining. It takes 1–2 h without heating.
2. OPTIONAL STEP: If the background level of CBB in the gel is high, replace the destaining solution containing 0.0001% (*w*/*v*) CBB R-250 to avoid excess destaining.

## 4. Expected Results and Discussion

CBB R-250 and G-250 are negatively charged dyes that contain two sulfonic (SO^3−^) groups. Although electrophoretic destaining has been previously demonstrated with a destaining solution containing 5% (*v*/*v*) methanol and 7.5% (*v*/*v*) acetic acid, two hours of electrophoresis was required for complete destaining [2]. I investigated different experimental conditions to electrophoretically remove CBB dye from the gels and found that the addition of glycine greatly accelerated the mobility of the CBB dye. It has been reported that the p*K*a values of CBB G-250 are 1.15 and 1.82 [17]. The pH value of the 10% (*v*/*v*) acetic acid solution was 2.2. By the addition of 100 mM glycine, the solution pH increased to 2.6. At a higher pH, the relative negative charge of the CBB dye and its mobility to the anode may increase. The glycine would also act as a counter ion of acetate and affect the electric field strength.

**Figure 1:**
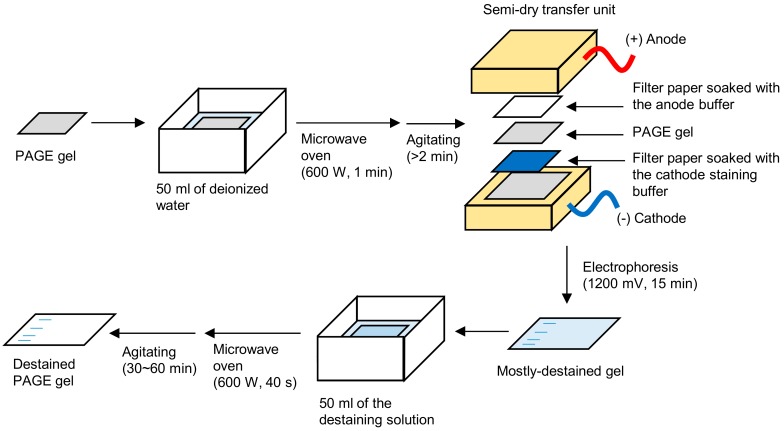
The procedure for the electrophoretic-staining/destaining method. PAGE: polyacrylamide gel electrophoresis.

To compare the detection sensitivity of proteins, *E. coli* lysate, molecular weight marker, and *E. coli* molecular chaperone GroES (10 kDa) and GroEL (58 kDa) were used as control proteins. The loaded protein amounts of GroES and GroEL were serially decreased to measure the detection limit. The samples were analyzed by SDS-PAGE, and the gels were stained and destained by the electrophoretic-staining/destaining method (electrophoretic method, hereafter) or the conventional method. The detailed protocols are described in the Experimental Design and Procedure sections. In this method, ethanol was used in the staining/destaining solutions instead of methanol. The overall experimental setup is shown in Figure 1. 

**Figure 2:**
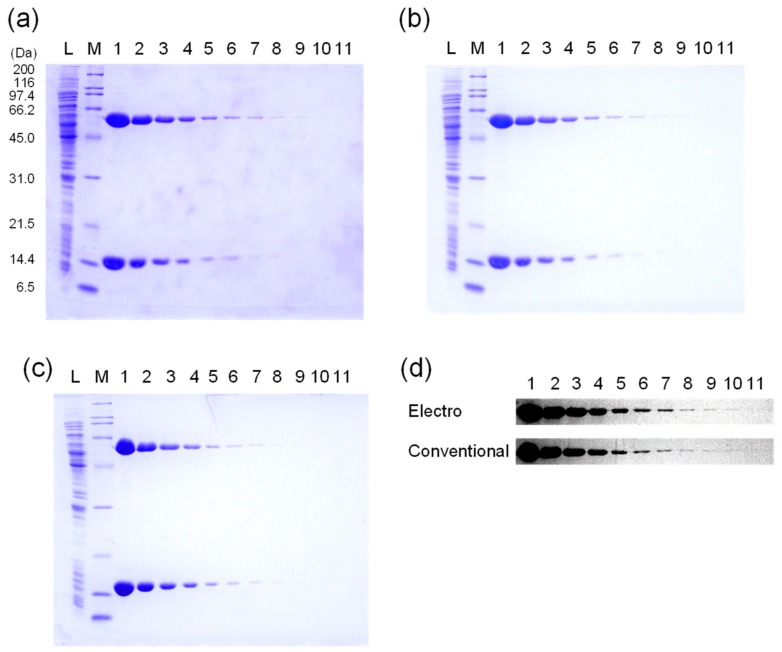
Comparison of the staining/destaining methods. Lane L, *E. coli* lysate; M, molecular weight marker; 1, 5 µg; 2, 2 µg; 3, 1 µg; 4, 0.5 µg; 5, 0.2 µg; 6, 0.1 µg; 7, 0.05 µg; 8, 0.02 µg; 9, 0.01 µg; 10, 0.005 µg; 11, 0.002 µg of *E. coli* molecular chaperone GroES (10 kDa) and GroEL (58 kDa). (**a**) A mostly-destained gel after the electrophoretic-staining/destaining method. (**b**) The gel of (**a**) destained with the secondary destaining method. (**c**) The gel stained by the conventional staining/destaining method. (**d**) The high contrast images of the GroEL bands (upper panel, the electrophoretic method; lower panel, the conventional method).

The gels, after the electrophoretic method and after secondary destaining, are shown in Figure 2a,b, respectively. After the electrophoretic method, most of the protein bands were visible, but the background level of CBB dye was still high. Residual CBB was completely destained after heating of the gel in the destaining solution by a microwave oven and subsequent agitation at room temperature for ~30 min (Figure 2b). The gel stained by the conventional staining/destaining method is shown in Figure 2c. The high contrast images of the GroEL bands stained by the electrophoretic method and the conventional method are shown in Figure 2d. For the lower amounts of GroEL (less than 0.1 µg (lane 6–11)), the band intensities obtained by staining with the electrophoretic method were higher than those stained with the conventional method.

**Figure 3:**
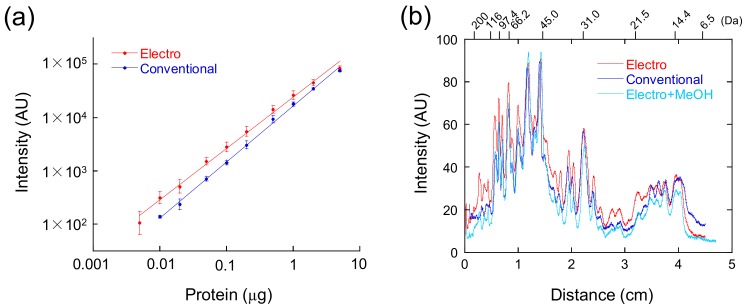
Sensitivity comparison. Red, the electrophoretic method; blue, the conventional method; cyan, conventional destaining after the electrophoretic method. (**a**) Correlation between the band intensities of GroEL and the loaded protein amounts. The average intensities and standard deviations from three independent measurements were plotted. (**b**) The profile of the band intensities of the *E. coli* lysate (lane L in Figure 1) compared to the distance from the top of the gel. The positions of the molecular weight markers are shown in the upper axis of the plot.

The correlation between the band intensities and loaded amounts of protein is plotted in Figure 3a. The band intensities of GroEL, stained by the electrophoretic method and the conventional method, were linearly correlated with the loaded protein amounts. The detection limit of the electrophoretic method (5 ng) was lower than that of the conventional method (10 ng) (Figure 3a). The band intensities of the *E. coli* lysate, stained by the electrophoretic method and the conventional method, were plotted (Figure 3b). At the high (>116 kDa) and middle molecular weights (~21.5 kDa), the band intensities from staining with the electrophoretic method were higher than those observed with the conventional method. The low sensitivity of the conventional method is caused by excess destaining. The band profile of the gel, destained by the conventional destaining solution after the electrophoretic method, was similar to that of the conventional method (Figure 3b). Thus, the destaining solution containing ethanol is better than the solution containing methanol to prevent excess destaining. 

## 5. Conclusions

Although this electrophoretic method requires more time than the rapid protein staining/destaining systems sold commercially, this method is cost-effective. High current power supplies are relatively expensive (~$1000), but each staining costs less than $0.5 per gel. This method is safer than other modified staining methods, as the hazardous methanol has been replaced with ethanol, and only one exchange of a hot solution is necessary. Ethanol in the destaining solution is also beneficial for increased sensitivity. Although secondary destaining is necessary for complete destaining, mostly-destained gels can be obtained in 30 min. This feature is useful for making rapid decisions for follow-up experiments. Furthermore, this method is more sensitive than the conventional method and comparable to the modified, rapid Fairbanks Coomassie stain method [9,10]. Therefore, this method is superior in speed, safety, low cost, and high sensitivity.

## 6. Reagents Setup

Acrylamide, bis-acrylamide, SDS, and glycine were purchased from Nakarai Tesque (Kyoto, Japan, Cat. No.: 10319-95, 22402-02, 31606-75, and 17109-35, respectively). CBB R-250 was purchased from Sigma-Aldrich (St. Louis, MO, USA, Cat. No.: B7920). Methanol, ethanol, and acetic acid were of extra pure reagent grade purchased from FUJIFILM Wako Pure Chemical (Osaka, Japan, Cat. No.: 137-01823, 059-00477, and 017-00251, respectively). Other reagents were of guaranteed reagent grade. The broad range molecular weight protein marker was purchased from Bio-Rad (Hercules, CA, USA, Cat. No.: 1610317). Purified *E. coli* molecular chaperones GroEL and GroES were used as protein standards [18]. The *E. coli* lysate was prepared as follows. The Top10 strain was cultured in 2× YT medium at 37 °C for 5 h. The cells, harvested from 1.5 mL of medium, were resuspended in 200 µL of TE buffer (20 mM Tris-HCl, pH 7.5, and 1 mM EDTA) and disrupted by sonication. The cleared lysate, prepared by centrifugation at 20,000× *g* for 10 min, was used as the *E. coli* lysate.
